# Climate controls on erosion in tectonically active landscapes

**DOI:** 10.1126/sciadv.aaz3166

**Published:** 2020-10-16

**Authors:** B. A. Adams, K. X. Whipple, A. M. Forte, A. M. Heimsath, K. V. Hodges

**Affiliations:** 1 School of Earth Sciences, University of Bristol, Bristol, UK.; 2 School of Earth and Space Exploration, Arizona State University, Tempe, AZ, USA.; 3 Department of Geology and Geophysics, Louisiana State University, Baton Rouge, LA, USA.

## Abstract

The ongoing debate about the nature of coupling between climate and tectonics in mountain ranges derives, in part, from an imperfect understanding of how topography, climate, erosion, and rock uplift are interrelated. Here, we demonstrate that erosion rate is nonlinearly related to fluvial relief with a proportionality set by mean annual rainfall. These relationships can be quantified for tectonically active landscapes, and calculations based on them enable estimation of erosion where observations are lacking. Tests of the predictive power of this relationship in the Himalaya, where erosion is well constrained, affirm the value of our approach. Our model allows estimation of erosion rates in fluvial landscapes using readily available datasets, and the underlying relationship between erosion and rainfall offers the promise of a deeper understanding of how climate and tectonic evolution affect erosion and topography in space and time and of the potential influence of climate on tectonics.

## Teaser

Rainfall rates set the scaling of relief and river incision in tectonically active landscapes.

## INTRODUCTION

The capacity of climate to influence tectonics has been of growing interest for over a century (*1*), but the debate has intensified recently as datasets and models designed to test this relationship have emerged. While it is easy to appreciate how rising mountain peaks might affect local climate and atmospheric circulation, the processes by which climate might influence rock uplift are less intuitive. Can climate-driven erosion trigger enhanced rock uplift via a combination of isostasy, changes in crustal rheology, and evolution of fault-system dynamics (*2*)? Before answering such questions, the fundamental connection between climate and erosion must be established.

The nature of correlations among climate, topography, and erosion rate is central to resolving the elusive question of whether climate and tectonics are dynamically coupled (*3*–*5*). Given the broad implications and fundamental nature of this problem, studies with a range of approaches and scope have been carried out. While global studies have provided important insights, they have not demonstrated a dependence of erosion rate on rainfall conclusively due to many covarying and potentially confounding variables that could not be isolated (*6*–*8*). Ferrier *et al*. (*9*) removed most of these confounding issues by focusing on the Hawaiian island of Kaua’i. However, they also removed the landscape controls associated with active rock uplift in doing so. This may be problematic because nonlinearities associated with thresholds of erosion and bedload transportation interacting with the stochastic distribution of storms are critical to the link between climate and erosion (*5*). The findings in Ferrier *et al*. (*9*) imply that erosion is linearly related to fluvial relief and nonlinearly related to rainfall, but global compilations suggest that erosion is nonlinearly related to fluvial relief with no clear dependence on rainfall (*6*, *10*). This disparity implies the need for a different approach to exploring how climate influences the relationship between erosion rate and topography and, therefore, potentially tectonics.

This study complements and improves upon previous work by combining the range of relief, rainfall, and erosion rates usually found only in a global study with the careful curation of data to ensure that only truly comparable, quasi-equilibrium catchments are considered that is usually only possible in local studies. We do not separate the data by author or study, only by attributes of the catchments. We begin by showing clear relationships among observations of rainfall, fluvial relief, and erosion that are consistent with, but independent of, river incision theory. We then demonstrate how well these observations are described by the familiar stream-power model (*11*). We find that the relationship between fluvial relief and erosion rate is nonlinear, but linearly modulated by mean annual rainfall. The observed nonlinear relationship between erosion and fluvial relief has substantial implications for the strength of coupling between climate and tectonics around the globe and is consistent with the expected influence of erosion thresholds interacting with a stochastic distribution of floods, two factors that are ubiquitous in nature.

### Observations from the Himalaya

To circumvent possible confounding factors in global studies, we compile a large but carefully curated dataset of new and published erosion rates from a single mountain range. Our compilation is restricted to catchments in tectonically active settings with morphologies suggestive of spatially uniform erosion rates. Selected catchments have drainage areas >9 km^2^ [to ensure thorough sediment mixing (*12*)] and are free of substantial glacial influence. They exhibit a wide range of fluvial relief but a narrow range of rock properties. Rainfall varies widely among the catchments. This is the largest dataset compiled to date (*N* = 142) that includes only truly comparable, quasi-equilibrium catchments (here defined as river networks whose channel profiles are well graded, implying that they do not record any temporal or spatial changes in erosion rate).

**Fig. 1:**
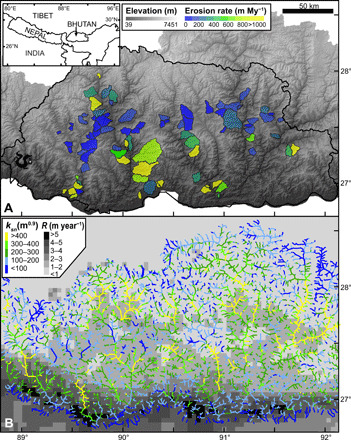
Topography, erosion, and precipitation in the Bhutan Himalaya. (**A**) Digital elevation model overlaid by new and previously published cosmogenic nuclide, basin-averaged erosion rates (*13*–*15*). Black line denotes the border of Bhutan (see inset). (**B**) Mean annual rainfall (*R*) data (*43*) overlaid by channel steepness (*k_sn_*) data.

We focus on testing the sensitivity of the relationship between fluvial relief and erosion rate to spatially variable climate in the Bhutan Himalaya, which has quickly become one of the most densely sampled mountain ranges for detrital cosmogenic nuclide erosion rates (Fig. 1A) (*13*–*15*). We have focused on this region because of the abundance of erosion rates from quasi-equilibrium basins where a broad range of erosion rates and rainfall rates is sampled across a broad spectrum of fluvial relief. New (see table S1) and previously published erosion rates from quasi-equilibrium, unglaciated catchments in Bhutan range between 22 and 3670 m My^−1^ (Fig. 1A) (*13*–*15*). Mean annual rainfall (*R*) ranges between 0.72 and 5.9 m year^−1^ (Fig. 1B) within these catchments. The highest rainfall rates, however, only occur in a narrow band near the foreland of the range. Most of Bhutan (including most of our sample locations; fig. S1) receives less than 2 m year^−1^ of rainfall annually. To more evenly sample across the range of mean annual rainfall, we also incorporate samples from central-eastern Nepal, which brings to the dataset more samples from high rainfall areas (0.99 to 4.2 m year^−1^) that span a wide range of relief and erosion rates (69 to 2122 m My^−1^) (*16*–*18*). Our aim for incorporating these data in our analysis is to achieve greater data diversity—rivaling that of previous global studies—while preserving the ability to firmly constrain key variables in a manner only possible in a local study. With this dataset, we can test the null hypothesis of a single relationship between topographic relief and erosion, which would predict that variable rainfall rates across the region would have little or no influence on erosion rates.

### River incision theory

To assess the influence of rainfall on the relationship between fluvial relief and erosion rates, we build on the classical stream-power river incision model, which can be written in terms of drainage area or discharge (*11*, *19*)

$$E=K•A^{m}•S^{n}$$ (1a)

$$E=K_{\mathrm{lp}}•Q^{m}•S^{n}$$ (1b)

$$K=K_{\mathrm{lp}}•R^{m}$$ (1c)

where *E* is the erosion rate (m year^−1^); *K* (units depend on *m*, when *m* = 1, the units are m^−1^ year^−1^) is the coefficient of erosion, often referred to as erosional efficiency, which encapsulates the influence of environmental conditions such as climate, lithology, and incision process (e.g., abrasion and plucking) (*19*); *A* is the drainage area (m^2^); *R* (m year^−1^) is the rainfall rate averaged over *A*; *Q* is the stream discharge (*A•R*, m^3^ year^−1^); *S* is the channel slope (dimensionless); and *m* and *n* are dimensionless constants related to channel incision processes, hydraulic geometry, basin hydrology, and runoff variability (*11*, *19*, *20*). *m* is the same in Eqs. 1a and 1b because the relationship between *Q* and *A* is assumed to be linear. *K_lp_* is a coefficient (m^−2^, for *m* = 1) that encompasses the effects of bedrock erodibility, channel geometry and roughness, incision process, and sediment flux but is independent of climate. The influence of mean annual rainfall, often subsumed by *K* (Eq. 1a), is treated explicitly in Eqs. 1b and 1c.

In quasi-equilibrium landscapes, stream-power model predictions match the empirical observation that erosion rates scale as a power function of channel slope and drainage area (*21*), and channel slopes are inversely related to drainage area (*22*, *23*). When local channel slopes are normalized for the nonlinear, downstream increase in drainage area, the resulting metric, normalized channel steepness (*k_sn_*, m^0.9^) allows the comparison of the relief of river channel regardless of the magnitude of the areas they drain

$$k_{\mathrm{sn}}=A^{\theta }•S$$ (2)

where θ is a dimensionless constant that measures the concavity of a longitudinal river profile (*22*, *23*). We find that θ = 0.45 describes the concavity of quasi-equilibrium river channels in Bhutan based on regressions of slope-area data (*24*); similar values have been used in Nepal (*18*).

Channel steepness is a robust, purely geometric measure for understanding the importance of spatial changes in channel slope, or channel relief, that can be measured without a priori knowledge of specific climate, lithology, or incision processes. Channel steepness can be calculated from topographic data where local channel slopes can be measured, and θ can be estimated from regressions of *S* and *A* (*25*), or regressions of elevation and ∫*A*^-θ^ (∫A^-θ^ is referred to as χ) (*26*). In quasi-equilibrium landscapes with spatially uniform lithology, climate, and rock uplift conditions, plots of elevation and χ are linear (see fig. S2), and the concavity is equal to the ratio of *m* and *n* from the stream-power model (i.e., θ = *m*/*n*) (*19*). Because discharge events are distributed in space and time, and the shear-stress thresholds required to initiate sediment transport or detach bedrock from a river bed are large, a nonlinear relationship between erosion rate and channel steepness is expected under quasi-equilibrium conditions (*10*, *27*), consistent with observations of river channels in tectonically active settings around the globe (*6*, *10*, *27*, *28*).

The channel steepness index is measured from topographic data alone (Eq. 2) and carries no specific climatic information. To incorporate climatic data, we calculate a channel steepness metric based on a simple proxy for discharge (*Q*)—the product of drainage area and mean upstream rainfall (Eq. 1b) where

$$k_{\mathrm{sn}}‐q=Q^{\theta }•S$$ (3)

The result is an enhanced channel steepness index we refer to as *k_sn_-q* (see Materials and Methods). Analogous methods have been used to calculate specific stream power and channel steepness (*18*, *29*, *30*). Our *k_sn_-q* metric is a scaled version of the well-known *k_sn_* metric; therefore, it has a similar response to spatial changes in slope and choice of θ [see (*31*) for more discussion]. The calculation of *k_sn_-q* combines the robust, empirically based channel steepness metric (Eq. 2) with the process-based theory of the stream-power model (Eq. 1). This can also be seen by substituting Eqs. 2 and 3 into Eq. 1 and solving for *k_sn_* and *k_sn_-q* to find

$$k_{\mathrm{sn}}=K^{-1/n}•E^{1/n}$$ (4a)

$$k_{\mathrm{sn}}={\left(K_{\mathrm{lp}}•R^{m}\right)}^{-1/n}•E^{1/n}$$ (4b)

$$k_{\mathrm{sn}}-q={K_{\mathrm{lp}}}^{-1/n}•E^{1/n}$$ (4c)

The theory surrounding the stream-power model and the channel steepness index makes a few important predictions. First, there need not be any correlation between climate metrics and erosion rates in quasi-equilibrium landscapes. On the basis of the definition of quasi-equilibrium (*32*), erosion rates will approximately equal rock uplift rates regardless of the local climate. In quasi-equilibrium landscapes with uniform rock uplift, any spatial variation in climate will instead be reflected in the topography (*16*, *33*, *34*). Therefore, provided uniform rock erodibility, and regardless of spatial variations in rock uplift rate, any influence of climate on erosion should be expressed in the relationship between topography (e.g., *k_sn_*) and erosion rate (*5*). The sensitivity of *k_sn_* − *E* relations to rainfall allows us to quantify the influence of climate and, thus, to test the predictive capacity of the stream-power model. For example, if incorporating rainfall into the stream-power model (i.e., *k_sn_-q*) adequately captures the influence of climate, data collected across a wide range of topography, rock uplift rates, and rainfall sample data should collapse to a single *k_sn_-q* – *E* relationship, provided that other factors influencing *K* are invariant (i.e., a single value of *K_lp_*). The null hypothesis of a single relationship between topographic relief and erosion would predict that finding a clear *k_sn_-q* – *E* relationship would fail and that the *k_sn_-q* – *E* relationship would likely be more scattered than the *k_sn_* – *E* relationship.

To explore how channel steepness varies as a function of erosion rate, we regress observed data using a power-law relationship of the form (see Materials and Methods)

$$k_{\mathrm{sn}}=C•E^{\Phi }$$ (5)

where *C* is the power-law coefficient and Φ is the exponent. The form of Eq. 5 is consistent with findings around the world (*6*, *27*, *28*, *35*) and is independent of, but consistent with, the stream-power model. All the information contained in Eq. 5 is purely geometric including the geometry of landscapes and the shape parameters of regression curves. However, by combining Eq. 5 with Eq. 4a, we can show that in terms of the stream-power model

$$C=K^{-1/n}$$ (6)

$$\Phi =n^{-1}$$ (7)

Thus, the results of our regression analysis are fundamentally linked to the physics of river incision and can be described by the stream-power model.

## RESULTS

### Statistical analysis of observations

**Fig. 2:**
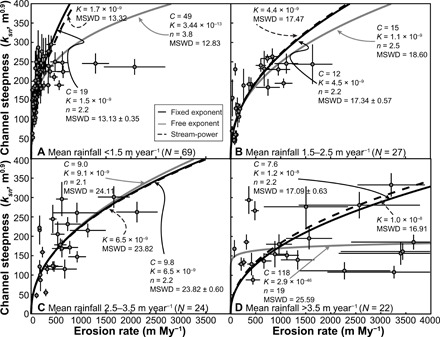
Best-fit relationships between channel steepness (*k_sn_*) and erosion rate for different rainfall bins. In all plots, the solid black and gray curves denote relationships as calculated by the least-squares estimation regression using a fixed and free exponent (*n*), respectively. The dashed gray curve is the solution for the stream-power model (Eq. 1b) given the rainfall bin centers, and a *K_lp_* value of 2.2 × 10^−9^ m^−2^. (**A**) Basins that receive less than 1.5 m year^−1^ annual rainfall. (**B**) Basins that receive between 1.5 and 2.5 m year^−1^. (**C**) Basins that receive between 2.5 and 3.5 m year^−1^. (**D**) Basins that receive more than 3.5 m year^−1^. Error bars represent 2σ uncertainties. *N* is the number of basins in each rainfall bin.

As a first approach to assessing how channel steepness responds to variation in mean annual rainfall, while also accounting for spatial variation in erosion rates, we performed regressions on four rainfall bins with similar size and spacing (<1.5, 1.5 to 2.5, 2.5 to 3.5, and >3.5 m year^−1^) so as not to overinterpret how well the influence of rainfall on the erosion coefficient can be resolved and to ensure sufficient data in each bin (Fig. 2). In our regressions, *C* and Φ could both vary (Eq. 5), and we also performed regressions with a fixed Φ (see table S2 for regression statistics). To assess the goodness of fit of our regressions to our observed data when the uncertainties in both variables are considered, we calculated the mean square weighted deviation (MSWD), where the MSWD ideally should be within the range 1 ± 2σ (see Materials and Methods) (Fig. 2). While fixing Φ may appear restrictive, doing so allows us to analyze comparable *C* values from different rainfall bins. In addition, we find that fixing Φ to a reasonable value of *n*^−1^, where *n* = 2.2, produces slightly better fits to our observed data (i.e., lower MSWD). We suggest that this effect is created by the presence of outliers in our dataset, where a few anomalously high erosion rates can drive best-fit analyses to more nonlinear relationships when the exponent is a free parameter. The *C* values found through regressions with fixed Φ values demonstrate that *K* increases with increasing mean annual rainfall, consistent with but independent of Eqs. 1a and 1c.

**Fig. 3:**
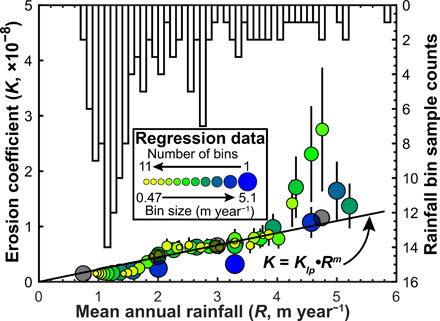
The effects of rainfall bin size and center on erosion coefficient calculations. Colored points show the resulting *K* values from regressions of binned sample data, where *n* = 2.2. These regressions are not influenced by *m* or *K_lp_*. The black line is derived from the stream-power model (Eq. 1c), where *m* = 1 and *K_lp_* = 2.2 × 10^−9^. An inverted histogram (calculated with 0.1 m year^−1^ bins) shows the distribution of mean annual rainfall (*R*) sample data analyzed in this study. Gray circles mark the four bins used in Fig. 2.

To explore the relationship between *K* and mean annual rainfall recorded in our observations more rigorously, without knowing how data would or should cluster, or introducing bias, we parsed our data into rainfall bins of varying size. We divided our sample mean annual rainfall range (0.72 to 5.9 m year^−1^) using 11 different bin sizes varying from 0.47 to 5.1 m year^−1^ (see Materials and Methods for details), yielding 66 different bins (Fig. 3), and performed a regression on each binned dataset. Figure 3 shows the comparison of *K* values based on the regressions of data in each rainfall bin (colored circles) using a fixed *n* = 2.2 and Eq. 6. While scatter in the regression data is expected, *K* can mostly be described as a continuous and near-linear function of rainfall as predicted by Eq. 1c, provided that there are enough data points in each bin. For this study, we require at least seven data points in each bin to consider the result robust. This is a conservative estimation designed to ensure robust regression results, but also to ensure that our results are not removing sparse, but vital information from the wetter bins. Some rainfall bins especially near the maximum rainfall values are much more variable due to the paucity of data at extreme rainfall values and the influence of possible outliers (see Fig. 2D). In small bins with centers above 4 m year^−1^, there are fewer samples in each bin, which resulted in poor regression fits due to small sample statistical issues. Be that as it may, our analysis demonstrates that based on observations alone, there is strong evidence that the erosional efficiency of rivers and rainfall in the Himalaya are directly linked through an apparently linear relationship.

Figure 2 illustrates the difficulty in constraining *n* from simply regressing binned data, which is critical to understanding how *K* changes with *R*, as illustrated in Fig. 3. Furthermore, although the regression results in Fig. 3 suggest *m* = 1, this analysis is for an assumed *n* = 2.2 (Φ = 0.45) only. To find plausible and compatible regional values of *n* and *m*, as well as *K_lp_*, which is critical for understanding spatially variable *K*, we turn to the stream-power model.

### Combining observations and the stream-power model

Combining our binning approach with the predictions of the stream-power model provides a mechanism for constraining critical parameters of landscape evolution based on observations of natural landscapes and reduces the sensitivity of regression analysis to possible erroneous data (see Materials and Methods for more details). To calculate regional best-fit values of *K_lp_*, *n*, and *m*, we test a range of possible *n* values within our mean annual rainfall bin framework. For each value of *n*, we regress each of the 66 rainfall bins with the exponent fixed, yielding a *K* value for each bin (see fig. S3 for examples of *n* = 1, 2.2, and 3). For each set of 66 bins, we calculate a mean *K_lp_* from the bin regressions using Eq. 1c and a value of *m* = θ•n, where θ = 0.45. With these possible *K_lp_*-*n*-*m* combinations, we use Eq. 4b to calculate predicted *k_sn_* values for our samples based on their observed *E* and *R* values. We take the regional best-fit parameter combination that minimizes the χ^2^ statistic (see Materials and Methods) between observed and predicted *k_sn_* values (fig. S4): *K_lp_* = 2.2 × 10^−9^, *n* = 2.2, *m* = 1 (Fig. 3).

**Fig. 4:**
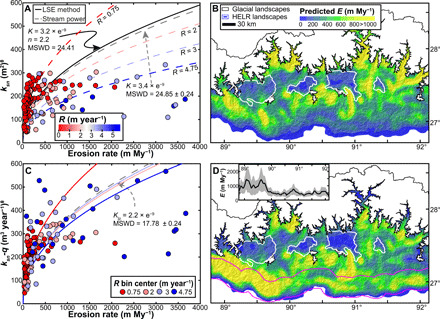
Relationships between channel steepness and erosion, and their predictive power. (**A**) Red and blue curves are the stream-power model curves from the four mean annual rainfall (*R*) bins in Fig. 2. Black curve is the regression of all data. The gray dashed line is the stream-power model calculated with the median sample rainfall (2.1 m year^−1^). (**B**) Map of erosion rates based on the distribution of channel steepness (Fig. 1B), *K* = 3.2 × 10^−**9**^ m^−**1**^ year^−1^ [calculated from the regression in (A) and Eq. 3a]. High-elevation, low-relief landscapes (HELR) are outlined in white. A white mask marks glacial landscapes above 4000 m above sea level. (**C**) Regressions of the four rainfall bins (red and blue curves) move toward the stream-power model (gray dashed) when *k_sn_-q* data are used. (**D**) Map of erosion rates based on the distribution of *k_sn_-q* and regional best-fit *n* and *K_lp_* values (see Materials and Methods). The 30-km-wide swath (magenta) shows the area sampled to calculate inset figure. Inset shows how the mean (black line) and SD (gray envelope) of erosion rates (*E*) near the foreland change with longitude.

With our regional best-fit parameters, we calculated predicted *k_sn_* – *E* relationships using the stream-power model, where *R* was set to the rainfall bin center as a means of comparison with our regressions (Fig. 2). We use the rainfall bin centers in the stream-power model in this calculation for simplicity. Figure 2 highlights that the predicted stream-power curves are close to the best-fit regression curves, and, in all cases, the stream-power model fits the data just as well as the regression (i.e., MSWD values are statistically indistinguishable). Figure 4A shows that these stream-power model curves predict most of the spread we observe in our dataset, suggesting that it can be explained by spatial changes in rainfall.

## DISCUSSION

### How well does the stream-power model predict observed erosion rates?

Our data and analysis show that the relationship between channel steepness and erosion is nonlinear (quantified by *n*). This is consistent with the findings from global datasets (*6*, *10*) and with the idea that thresholds of erosion and sediment transportation, as well as the stochastic distribution of storms, are critical to understanding the form and function of active mountain ranges and the possibility of climate-tectonic coupling. Our observations are, however, inconsistent with recent interpretations from Kaua’i (*9*), where data point to a linear relationship between fluvial relief and erosion rate. This disagreement may suggest a distinct set of controls on long-term river incision rates in tectonically active landscapes (i.e., the Himalaya), as compared to tectonically inactive landscapes (i.e., Hawai’i). Our findings suggest a strong limitation to the generality of the interpretation that stream power is linearly related to erosion. Moreover, we find a linear, rather than sublinear, relation between rainfall and the coefficient of erosion, *K*.

Although the nonlinearity of the of *k_sn_* – *E* relationship is expected to depend on runoff variability (*20*) and there is an expected negative correlation between runoff variability and mean annual rainfall (*36*, *37*), there is no evidence that *n* varies in a meaningful way as a function of rainfall in our dataset. This may not be unexpected because a recent study by Rossi *et al*. (*37*) shows that, in the contiguous United States and Puerto Rico, the correlation between mean runoff and its variability is weak for mean annual rainfall above 1.5 m year^−1^. Similarly, Scherler *et al*. (*38*) found minimal differences in runoff variability for Himalayan rivers across 800 km along strike of the range and spanning a range in mean annual rainfall of 1.5 to 4 m year^−1^. This suggests that only the driest rainfall bin in Fig. 2A would be expected to have a measurably lower *n* value than the other rainfall bins (*5*, *38*). However, the *n* value for our driest bin (<1.5 m year^−1^) in regressions with *n* as a free parameter is higher than other wetter bins, implying that *n* is not influenced by variability, or the regression technique is too heavily influenced by scatter in the data including possible high-erosion rate outliers to be able to resolve an influence of enhanced discharge variability in more arid climates.

When *n* can vary in the wettest bin (Fig. 2D), the best-fit regression produces a highly unlikely relationship (*n* = 19 and *K* = 3 × 10^−46^). This excessively nonlinear relationship is strongly influenced by a group of low-steepness, high-erosion samples. While these data have large uncertainties, they are likely accurate and corroborate independent interpretations of high erosion in southwestern Bhutan as evidenced by very young ages of low-temperature thermochronometers (*39*). It is possible that these low-relief, high-erosion landscapes may reflect a greater erodibility of rocks exposed in this part of Bhutan. A nearly sixfold increase in *K_lp_* (*K_lp_* = 1.2 × 10^−8^ m^−2^) would be required for the stream-power model to fit these data (fig. S5). Without more data from southwest Bhutan, it is not possible to rigorously test this idea.

When plotted together (Fig. 4A), the stream-power model curves from the four rainfall bins illustrate how rainfall can limit the channel steepness, and thus the overall relief of a mountain range, despite vigorous rock uplift and commensurate erosion rates. The spread in these curves provides a good representation of the dispersion that we should expect to see in our dataset regardless of the possibility of outliers, or complications of lithology, but simply as a function of spatial rainfall variation. While a regression curve for the entire dataset can be calculated, this curve would be misleading because it mixes different populations of data with likely different *k_sn_* – *E* relationships. Although a stream-power model curve based on the median rainfall value lies very close to the regression of all data (Fig. 4A), using the regression results to perform a regional analysis of erosion based on channel steepness, *n*, and a single *K* value, assumes a homogenous rainfall rate across all landscapes (Fig. 4B, see Materials and Methods), which leads to erroneous predicted erosion rates where rainfall rates deviate from 1 m year^−1^. Figure 4B demonstrates that the null hypothesis that there is a single relationship between topographic relief and erosion does not hold for our studied region of the Himalaya. We find instead that the relationship is dependent on rainfall, and the effect of variable rainfall is reasonably accounted for by the coefficient of erosion.

Our finding that *K* increases monotonically with mean annual rainfall (Figs. 2 and 3) suggests that the influence of rainfall on erosion rates could be effectively captured in *k_sn_-q*, the enhanced channel steepness metric based on a proxy for discharge rather than simply drainage area introduced above (Eq. 3). When *k_sn_-q* is plotted against observed erosion rates (Fig. 4C), the scatter in the data is much reduced, and the nonlinear relationships of individual rainfall bins converge toward the stream-power curve, which is a function only of our best-fit *n* and *K_lp_* parameters (Eq. 4c). Because it embeds the influence of mean annual rainfall, a predicted erosion rate map based on *k_sn_-q* (Fig. 4D) produces a more complete picture of the regional pattern of erosion as compared with the map generated using *k_sn_* (Fig. 4B). Predictions of erosion rates based on topography alone (i.e., interpreting a map of channel steepness) can lead to large over- and underestimations across a region with variable rainfall (fig. S6).

### Implications of the erosion pattern inferred in the Bhutan Himalaya

If our analysis sufficiently accounts for the spatial influence of rainfall on erosion rates, our erosion rate map provides insight into the deformation of the eastern Himalaya, albeit with some important caveats. While our sampled river basins span a wide variety of rock types (*40*), we are unable to discern any effects of lithology within our analysis (see fig. S7). This implies, as others have suggested, that there may be no fundamental difference in the erodibility of different Himalayan rocks (*41*) or tectonostratigraphic units (*16*), or that recovering the influence of rock type would require a more specific sampling strategy. Our erosion maps are produced using modern topography and rainfall, both of which have likely varied over time (*14*, *42*)—at best, our map captures the pattern of modern erosion rates. Mean annual rainfall rates are based on calibrated remote sensing data averaging over 12 years (1998 to 2009) (*43*). The time scales recorded in the topography of the Himalaya and cosmogenic erosion rates are considerably longer. However, our landscapes do not record any perturbations that would indicate a climate change large enough to alter the topography or erosion rates, and thus, we conclude that the modern rainfall data are likely reflective of long-term patterns and coupled with our erosion and topographic observations.

We acknowledge that our analysis does not directly incorporate the influences of snowfall. Because the vast majority of precipitation in Bhutan arrives as rain in the summer monsoon season, and our basins are from lower-elevation, nonglaciated portions of the landscape, and only large floods exceed erosion and transport thresholds, we do not consider snow melt an important factor in controlling channel steepness. Our estimated erosion rates are not accurate in landscapes where glaciers have modified topography (see glacial landscapes in Fig. 4, B and D) in a manner that is not consistent with the stream-power model (*44*, *45*), and erosion rates cannot be directly interpreted as reflecting modern rock uplift rates in landscapes that include transient signals of tectonic change (*14*, *46*). However, once these transient landscapes are masked, leaving only quasi-equilibrium portions of the region, robust spatial patterns in the predicted erosion rates can be assessed and rock uplift rates can be inferred. Previous studies have suggested that basin-averaged erosion rates can reveal rock uplift patterns and, thus, underlying tectonic architecture in Bhutan (*15*). However, we assert here that such interpretations are only valid in quasi-equilibrium landscapes, and caution is warranted as large portions of Bhutan are in disequilibrium, where erosion rates may be up to 10 times slower than rock uplift rates (*14*). While our maps are based on erosion rates with millennial-scale integration times, the observed patterns may have persisted for much longer. Given that most landscapes adjust to changes in erosion rate on million-year time scales (*47*), it is likely that our cosmogenically derived erosion rates reflect erosion rate patterns established and sustained on a million-year time scale.

The predicted erosion rates in Fig. 4D exhibit a notable change around 90°E near the range front (see inset). Because our erosion rates are roughly equal to rock uplift rates in our quasi-equilibrium landscapes, the exhibited dichotomy must be created by spatial changes in tectonic velocities. The two most likely mechanisms are a change in convergence rate or change in fault geometry associated with the Himalayan sole thrust. It is possible that convergence rates change across Bhutan because there is a transfer structure within the Indian plate whereby slip rates east of ~90°E have been reduced due to some accommodation of Indio-Eurasian convergence across the Shillong Plateau. Alternatively, a change in the dip of the Himalayan sole thrust where the dip is greater west of ~90°E (i.e., lateral ramp in the sole thrust) could create the same pattern without requiring a change in convergence rate in the Bhutanese Himalaya. Transfer structures between the Himalaya and the Shillong Plateau have been proposed (*48*, *49*) based on geophysical evidence. Higher dip angles of the Himalayan sole thrust have been suggested in western Bhutan, as compared to eastern Bhutan, based on the inversion of low-temperature thermochronometers (*50*). However, our data cannot differentiate between these mechanisms, though it can accurately demarcate the important transition zone, which is critical for future studies.

## CONCLUSIONS

Our study of erosion rates in the Himalaya demonstrates that the stream-power model can be readily used to investigate the pattern in erosion rates when spatially variable, rainfall-dependent *K* values are incorporated into the analysis, and *K_lp_*, *n*, and *m* are well constrained, as done here for data in Bhutan and Nepal. Our findings are broadly consistent with the stochastic-threshold version of the stream-power river incision model that predicts a nonlinear (*n* > 1) relationship between erosion rate and channel steepness (*10*). The constraints our study places on the quantitative relationships between erosion, rainfall, and topography are fundamental to the integrated landscape evolution-mechanical models that are needed to thoroughly test the ideas of climate-tectonic coupling, and the thermo-kinematic models that are used to test long-term exhumation/landscape evolution histories. More generally, the calculation of rainfall-dependent *K* is critical for studying how a changing climate modifies Earth’s surface and the time scales over which the modification occurs.

There are several fundamental implications of the observed relationship between erosional efficiency, erosion, and fluvial relief. Climate moderation of channel relief in Bhutan is strong enough to obfuscate the dominant signal of erosion and rock uplift, as recorded in the topography. Although Bhutan boasts the steepest mountain front along the Himalayan arc (*51*), these mountains would be considerably steeper if not for the strength of the monsoon in Bhutan. Mountain ranges influenced by orographic precipitation patterns around the world likely experience the same effects. The coincidence of high rainfall rates and high rock uplift rates within many mountain ranges, including the Himalaya, may explain the proposed global limit to channel steepness (*8*). We suggest that the paucity of channel steepness data above 300 m^0.9^ in Fig. 4A is not a limit of fluvial relief or response but simply shows the maximum values that can be achieved given the current rock strength, rock uplift rates, and rainfall rates in the region. While our findings are based on exploring observations of quasi-equilibrium landscapes, they can be used in transient solutions of the stream-power model such that changes in erosion and topography could be modeled in response to temporal changes in mean annual rainfall and rock uplift rates.

## MATERIALS AND METHODS

### ^10^Be sample preparation

All samples were processed in the School of Earth and Space Exploration at Arizona State University, Surface Processes WOMBAT Laboratory. Dried sediment samples were sieved to 250 to 1000 μm and washed in tap water. Washed sediments were cleaned in a 1:1 solution of hydrochloric acid (HCl) and nitric acid (HNO_3_) at room temperature for at least 12 hours. Sediments were leached in a 5% hydrofluoric acid (HF) and HNO_3_ solution and rolled on heat for 24 hours. Feldspars and micas were floated off using a wetting technique. Minerals denser than 2.85 g cm^−3^ were removed via lithium polytungstate (LST) solution separation. During heavily liquid separation, water was added piecemeal to further separate target quartz from slightly denser and less dense lithic and nonquartz mineral fractions. During the quartz purification process, samples were leached at least five times with HF and HNO^3^ solutions on heated rollers. Each leach lasted 24 hours, and the final leach was for 7 days. Quartz separates were spiked with ^9^Be and digested with HF and HNO^3^. We removed interfering cations and anions using liquid chromatography techniques. Oxidized beryllium was mixed with a matrix of niobium and loaded into cathodes for analysis on an accelerator mass spectrometer at PRIME Lab, Purdue University. Beryllium isotope ratios (table S2) were referenced to the isotope ratio standards described by Nishiizumi and others (*52*).

### Basin-averaged erosion rate calculation

We follow the approach of Portenga and Bierman (*7*) to calculate basin-averaged erosion rates to capitalize on the protocols of the CRONUS online calculator (*53*). To accomplish this, we simplified the geometry of each basin to a single point by calculating an effective elevation, latitude, and longitude value of the entire basin. On the basis of the Shuttle Radar Topography Mission 30-m resolution digital elevation dataset, we calculated a scaled ^10^Be production rate based on the elevation and latitude of each pixel in each basin. To be internally consistent with the procedures of the CRONUS calculator, we calculated the production rate from spallation reactions using the scheme of Stone (*54*). We then calculated the mean of all total production rates within the basin and found the elevation and latitude values corresponding to this mean scaling factor, referred to here as the effective elevation and latitude of the basin. Effective latitude and elevation, centroid longitude, and calculated ^10^Be concentrations were entered into the CRONUS online calculator (version 2.3, accessed April 2018), assuming no topographic shielding (*55*). For CRONUS calculations, the following inputs were used: elevation flag = std, thickness = 1 cm, density = 2.7 g cm^−3^, Be standard = 07KNSTD, and Al standard = KNSTD. Because our effective latitude and elevation calculations are not time dependent, we report output CRONUS erosion rates based on the constant production rates determined by the models of Lal (*56*) and Stone (*54*) (table S2). We have recalculated erosion rates from previous studies in Bhutan and Nepal so that the rates in this study are internally consistent. However, we have not included samples from glaciated terrains, where drainage areas are less than 9 km^2^, or samples that were described as outliers by the authors of those studies to avoid the complication that these samples may represent in terms of quantifying erosion rates or topographic metrics.

### Channel steepness (*k_sn_* and *k_sn_-q*) calculation

Quasi-equilibrium longitudinal river profiles often have a form set by a power-law relationship between channel slope and drainage area (Eq. 2). We find that θ = 0.45 describes the concavity of quasi-equilibrium river channels in Bhutan and Nepal based on regressions of slope-area data (*24*), and thus, we used that value to normalize channel slope for the change in upstream drainage area—producing a normalized channel steepness (*k_sn_*). We made these calculations using TopoToolbox (*57*) and the TAK addon functions (*58*). To calculate our discharge-based channel steepness (Eq. 3), *k_sn_*-*q*, we used TAK to weight *A* by the mean annual rainfall as estimated from the Tropical Rainfall Measuring Mission dataset (*43*). We calculated area- and discharge-based channel steepness values for all positions where the accumulation area is greater than 1 km^2^. To make the *k_sn_* (Fig. 1B) and *k_sn_-q* maps, we used the TAK tool with the “trunk” smoothing option and a 6-km smoothing window for channels of Strahler order 4 and higher. For orders 1 to 3, the average smoothing length is dictated by tributary spacing and was 1.4 km.

### Regression calculations

To find the shape parameters for relationships between channel steepness and erosion rates, we carried out least-squares estimations (*59*). It is commonplace to log-transform power-law related data to use a linear, least-squares regression. While this may be convenient for data without associated uncertainties, it can be problematic for data with uncertainties, as these uncertainties are difficult to propagate in log space. Furthermore, the inclusion and improper weighting of uncertainties can create biased regressions within data that are nonuniformly distributed in *x* and *y*, or have uncertainties that scale with the mean value, both of which are true in our study. Our regression protocol was designed with three primary objectives: (i) incorporate uncertainties in *x* and *y*; (ii) equally weight uncertainties in *x* and *y* (i.e., ensure that high erosion rate data with higher uncertainties are not preferentially ignored); and (iii) quantify how well the model relationship calculated from the regression fits all observations used in the regression, including the influence of the uncertainties in *x* and *y*.

To accomplish these goals, we started with the equations of York (*59*) to perform least-squares estimations. We circumvent the issue of log-transforming uncertainties by using a Monte Carlo protocol, creating synthetic populations of *x* and *y* data pairs based on the mean values and associated uncertainties on those means. We then regressed these data with uniform, synthetic uncertainties in *x* and *y* (i.e., σ*_x_* and σ*_y_* = 1). These synthetic uncertainties equally weight each sample and ensure that the regression minimizes residuals orthogonal to the regression (*60*). Thus, the regression is not biased to one variable (i.e., *x* or *y*) that is inherently more precise than the other. With these synthetic populations, we calculate 10^6^ regressions on a randomly selected *x*-*y* pair from the synthetic distribution of each sample. The mean slope of the 10^6^ regressions yields the exponent of the nonlinear regression (Φ). After the slope exponent has been calculated, and in the case when it is prescribed, the best-fit preexponential coefficient (*C*) is calculated by regressing all 10^6^ synthetic points with the best-fit slope.

While it may be a common practice to use the coefficient of determination (*R*^2^) as a goodness-of-fit parameter for linear and nonlinear regressions, we do not find this statistic suitable for our needs. *R*^2^ does not include the influence of uncertainties of observations. Furthermore, the *R*^2^ value of data perfectly fit to a nonlinear model can exceed one (*61*), suggesting that high *R*^2^ values can be misleading (i.e., *R*^2^ values approaching 1 may be far from perfect). Instead of *R*^2^, we calculate the MSWD (*62*) of our modeled regressions. The MSWD is a form of the reduced chi-squared statistic)

$$\text{MSWD}=\frac{\Sigma \chi^{2}}{N-d}$$ (8)

where *N* is the number of observations, *d* is the degrees of freedom, and

$$\chi^{2}=\Sigma r^{2}W$$ (9)

where *r* is the residuals, and the weights (*W*) are determined by

$$W=\frac{1}{\frac{\partial F}{\partial x}\sigma_{x}^{2}+\frac{\partial F}{\partial y}\sigma_{y}^{2}}$$ (10)

where *F* is the function relating *x* and *y*, and σ*_x_* and σ*_y_* are the 1σ uncertainties on *x* and *y*, respectively. The 1σ uncertainty on the MSWD is given by (*62*)

$$\sigma =\sqrt{\frac{2}{N-d}}$$ (11)

For well-fit models, the MSWD should approach 1 ± 2σ (*62*).

### Calculation of best-fit *K_lp_*, *n*, and *m*

Our conceptual model suggests that the regressions of our observed erosion rates and channel steepness are fundamentally connected to the stream-power model, and therefore, we seek to evaluate how well the predictions of the stream-power model match our observations. We seek a method to determine the unknown variables within the stream-power model that satisfy our observations. One way of achieving this is to find values of *K*_lp_, *n*, and *m* that minimize the difference between *k_sn_* values predicted by the stream-power model (Eq. 4b) and observed *k_sn_* values. We set *m* = θ*•n*, where θ = 0.45 [see the “Channel steepness (*k_sn_* and *k_sn_-q*) calculation” section for discussion of θ]. Because the units of *K_lp_* depend on *m*, and thus on *n*, each value of *m* will be associated with a best-fit *K_lp_* value. To determine plausible *K_lp_* values for our dataset based on varying *m* and *n*, we use regressions of *E* versus *k_sn_* for catchments that have binned according to mean annual rainfall to determine *K* as a function of *R* and calculate *K_lp_*. Without imposing any constraints on the best-fit *n* value, or knowing a priori what are reasonable rainfall rate groupings within our dataset, we tested 21 different *n* values between 1 (linear *k_sn_* – *E* relationship) and 3 (highly nonlinear *k_sn_* – *E* relationship) across a distribution of possible rainfall-based subgroups. We created 66 rainfall bins of varying size and bin center that cover the range of rainfall values from 0.47 to 5.1 m year^−1^, based on observed values in Bhutan and Nepal.

For each *n* value, we calculated 66 regression-based *K* values (with a fixed exponent Φ *=* 1/*n* for each simulation), and then calculate *K_lp_* (*K_lp_* = *K/R^m^*; Eq. 1c) for each bin, where *R* was the bin center. We then calculated the mean *K_lp_* value for each tested *n* value for all bins with seven or more samples per bin using Eq. 1c. Using the resulting *K_lp_-n-m* triplets, we calculate predicted *k_sn_* values using Eq. 4b. Our best-fit *K_lp_-n-m* was found by reducing χ^2^ value between predicted and observed *k_sn_* values where

$$\chi^{2}=\Sigma \frac{{\left(k_{\mathrm{sn}}\;(\text{predicted})-k_{\mathrm{sn}}\;(\text{observed})\right)}^{2}}{\sigma \;{\left(k_{\mathrm{sn}}\;\text{observed}\right)}^{2}}$$ (12)

Similar to what has been found in many previous studies, a broad suite of combinations of *K_lp_*, *n,* and *m* yields plausible fits to channel steepness and erosion datasets (fig. S4) (*6*). The best-fit value *n* is 2.2, *m* is 1, and *K*_lp_ is 2.2 × 10^−9^ m^−2^. Our finding of *n* = 2.2 is in line with theory (*20*) and other studies (*6*, *10*, *27*), but we do not suggest that this represents a universal *n* value. We emphasize that using other *n* values around 2 would not change the conclusions or interpretations of this study.

### Predicted erosion rate map calculation

To produce the predicted erosion rate maps based on the stream-power model in Fig. 4 (B and D), we first created spatially continuous *k_sn_* and *k_sn_-q* maps, by averaging channel values over a 5-km-radius moving window. We then used these spatially continuous data and our best-fit parameters to calculate erosion rates at each pixel.

For the erosion rate map based on *k_sn_* (Fig. 4B), we calculated an erosion rate at each pixel using

$$E=K•{k_{\mathrm{sn}}}^{n}$$ (13)

where *K* = 3.2 × 10^−9^ m^−1^ year^−1^ and *n* = 2.2. We calculated *K* from the regression of all data (Fig. 4A), where *K* = *C^−n^*. For the erosion rate map based on *k_sn_-q* (Fig. 4D), we calculated an erosion rate at each pixel using

$$E=K_{\mathrm{lp}}•k_{\mathrm{sn}}-q^{n}$$ (14)

where *K_lp_* = 2.2 × 10^−9^ m^−2^ and *n* = 2.2. We only need to use *K_lp_* in the equation as *R* is included in *k_sn_-q.*

## Supplementary Material

aaz3166_SM.pdf

## SUPPLEMENTARY MATERIALS

Supplementary material for this article is available at http://advances.sciencemag.org/cgi/content/full/6/42/eaaz3166/DC1
