# Supplementary material for: Climate controls on erosion in tectonically active landscapes
Source: Sci Adv. 2020 Oct 16;6(42):eaaz3166. doi: 10.1126/sciadv.aaz3166 (PMC7567587; doi:10.1126/sciadv.aaz3166)
Supplement: aaz3166_SM.pdf [file aaz3166_SM.pdf]

Science Advances
AAAS

advances.sciencemag.org/cgi/content/full/6/42/eaaz3166/DC1

Supplementary Materials for

Climate controls on erosion in tectonically active landscapes

B. A. Adams*, K. X. Whipple, A. M. Forte, A. M. Heimsath, K. V. Hodges

*Corresponding author. Email: byron.adams@bristol.ac.uk

Published 16 October 2020, *Sci. Adv.* **6**, eaaz3166 (2020)
DOI: 10.1126/sciadv.aaz3166

This PDF file includes:

Figs. S1 to S7
Tables S1 and S2

Supplementary Materials

Supplementary Figures and Tables

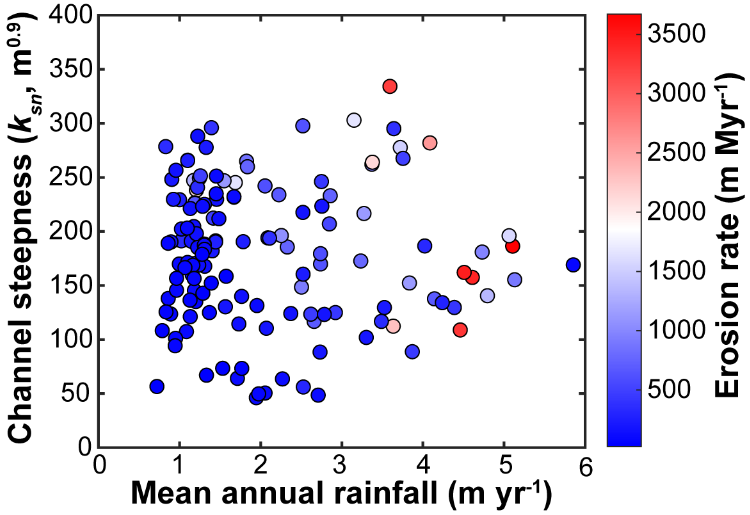

Fig. S1. Comparisons of mean annual rainfall, erosion rate, and channel steepness from the study area. Note that there is no single relationship that defines the dataset. However, a rough pattern emerges when considering the basin-averaged erosion rates.

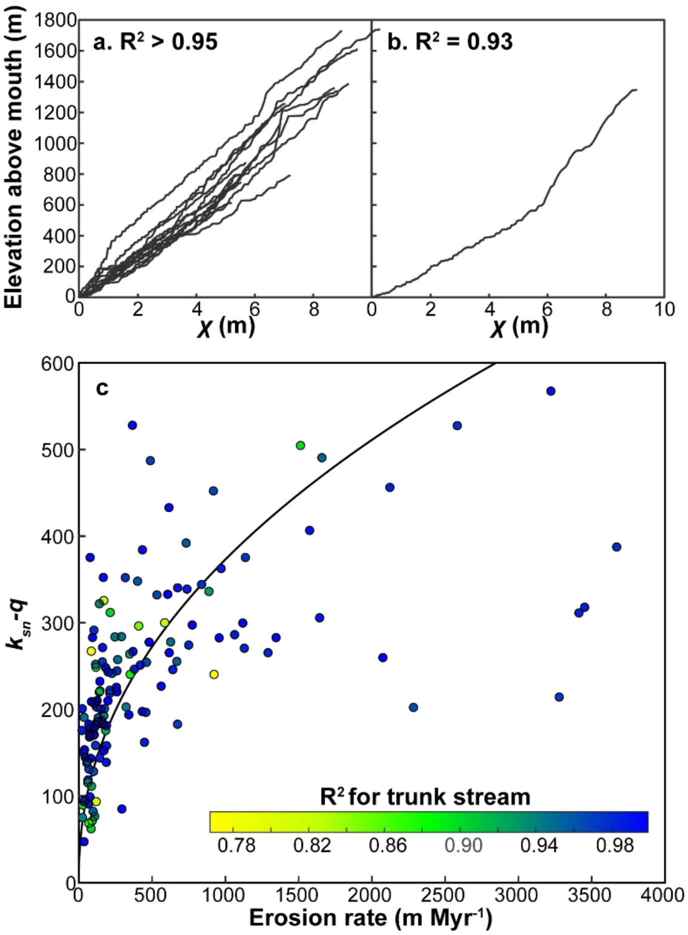

Fig. S2. Assessing quasi-equilibrium landscapes. a) χ plots of new sampled basins from Bhutan that exhibit an R^2 value greater than 0.95. b) χ plot of the new sampled basin from Bhutan that exhibits an R^2 value less than 0.95. c) Plot of all k_{sn}-q values and erosion rates for all sample data colored by R^2 value of the χ plot. The black curve represents our solution for the stream-power model where n is 2.2, m is 1, and K_{lp} is 2.2•10^{-9} m^{-2}. This solution does not change if we recalculate these values after having removed all basins with R^2 values lower than 0.95.

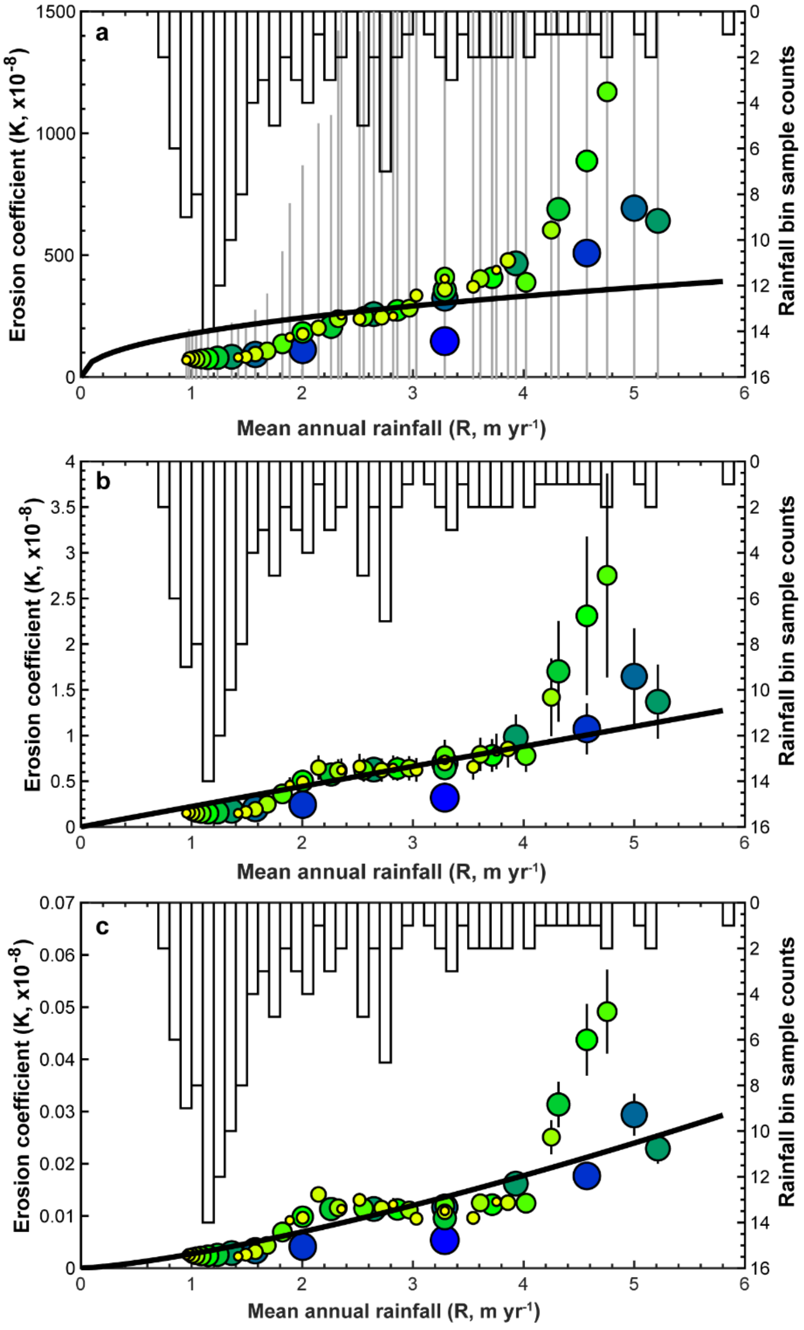

Fig. S3. The effects of varying *n* to find best-fit parameters. Colored points show the resulting *K* values from regressions of binned sample data where $n = 1$ (a), $n = 2.2$ (b), and $n = 3$ (c). The black line is derived from the stream-power model (equation 1c). An inverted histogram (calculated with 0.1 m yr^{-1} bins) shows the distribution of mean annual rainfall (*R*) sample data analyzed in this study. See text and Fig. 3 for more details. The units of *K* are dependent on *m* (equation 1c), which changes with *n*. The units of *K* in a), b), and c) are $m^{0.1}$ yr^{-1}, m^{-1} yr^{-1}, and $m^{-1.7}$ yr^{-1}, respectively. Note that the *K* values also fluctuate by orders of magnitude when n changes, and in the very unlikely case that $n = 1$ the uncertainties on the regression *K* values are very large.

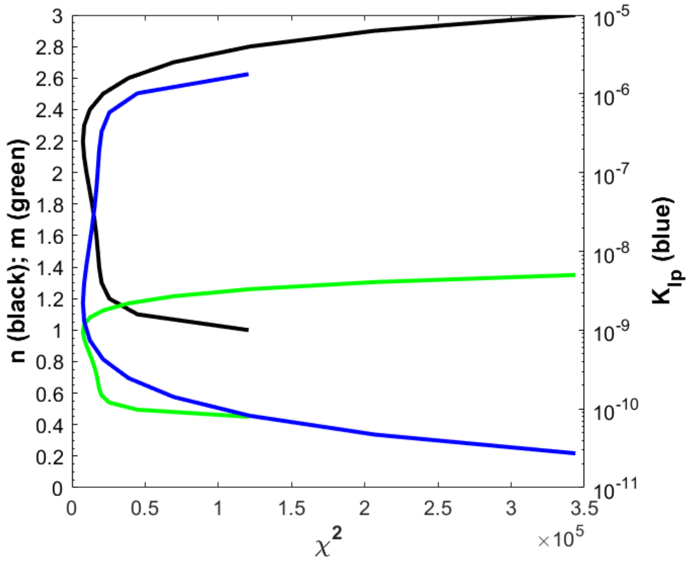

Fig. S4. χ^2 values calculated from different combinations of the slope exponent, *n*, and the partial coefficient of erosion, K_{lp}, and the area exponent, *m*. χ^2 is at a minimum where the K_{lp}-*n*-*m* triplet yields the smallest cumulative mismatch between predicted and observed k_{sn} values. Best-fit values are $n = 2.2$, $m = 1$, and $K_{lp} = 2.2 \cdot 10^{-9}$ m^{-2}.

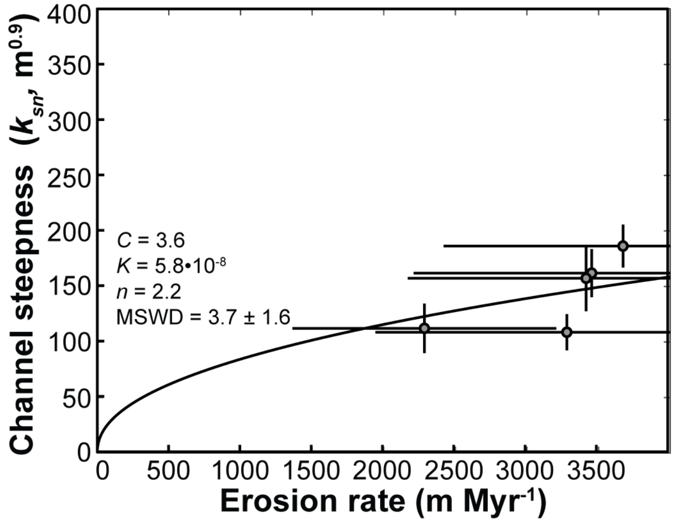

Fig. S5. Testing variable K_{lp} values in Bhutan. The stream-power law curve to fit the outlier low-relief, high erosion rate landscapes in southwestern Bhutan. Taken at face value, this regression suggests a K_{lp} value of $1.2 \cdot 10^{-8}$, which is ~6 times more erodible than the regional best-fit K_{lp}.

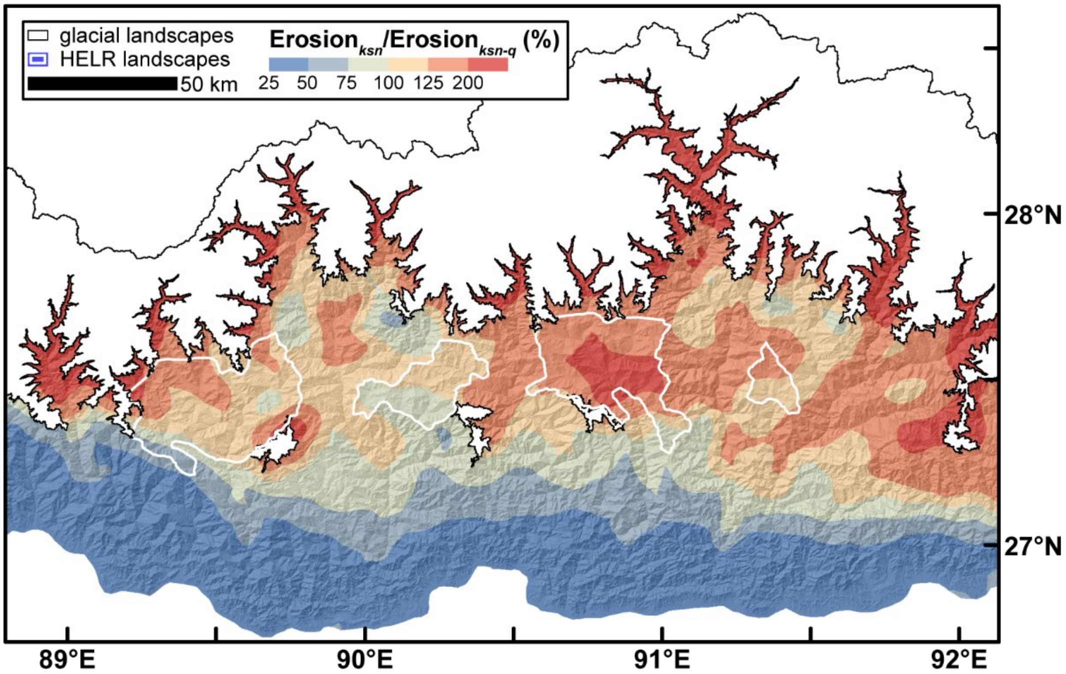

Fig. S6. Comparison of the erosion rates calculated from area-based channel steepness maps (Erosion$_{ksn}$) and the erosion rates calculated from the discharge-based channel steepness (Erosion$_{ksn\text{-}q}$). Note that in the wetter front ranges erosion rates based on area-based channel steepness leads to upwards of a 4-fold under prediction, and in the drier hinterland upwards of a 3-fold over prediction.

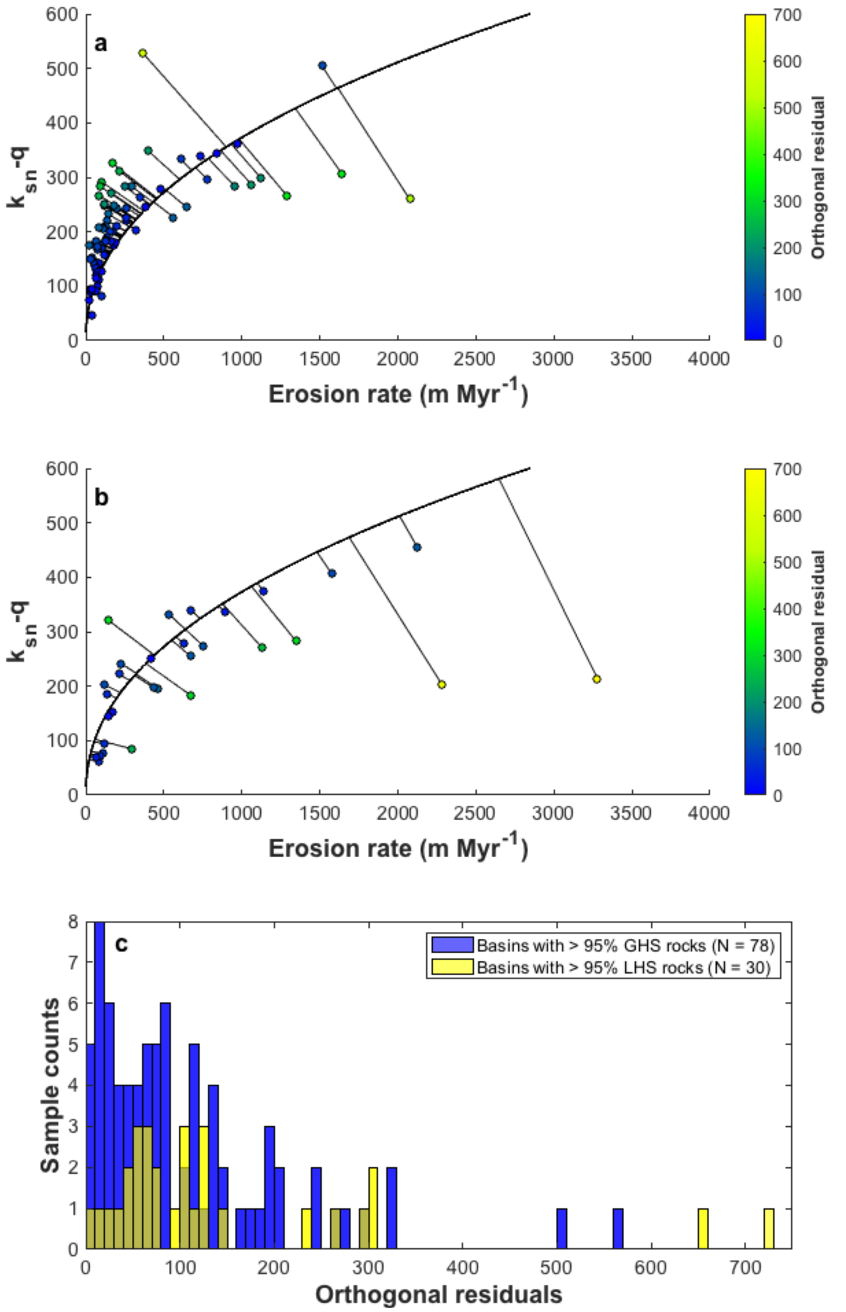

Fig. S7. Analysis of tectono-stratigrahic units of the Himalaya. a) Orthogonal residuals of observations from basin-averaged samples collected within the Greater Himalayan lithologies (upper amphibolite to granulite ortho- and para-gneisses) referenced to our best-fit stream-power model. b) Orthogonal residuals of observations from basin-averaged samples collected within the Lesser Himalayan lithologies (predominantly green-schist metasedimentary rocks). c) Distribution of orthogonal residuals for both units demonstrating that samples from the two units do not represent different populations. Therefore, there is no evidence for different K_{lp} values in these rock packages, consistent with findings of prior studies (see text for details). N is the number of samples represented in each unit.

Table S1. Laboratory and isotopic data for new Bhutan samples and blanks.

Sample Name	Latitude (°N)	Longitude (°E)	Elevation (m a.s.l.)	Quartz (g)	Be from spike (g)	$^{10}Be/^{9}Be$ from AMS	$^{10}Be/^{9}Be$ 1σ	Blank ^{10}Be (atoms)	Blank ^{10}Be 1σ (atoms)	Be Blank Used	[^{10}Be] (atoms/g)	[^{10}Be] 1σ (atoms/g)
BT09103	26.876629	90.266828	294	103.6220	1.80E-04	1.90E-13	2.78E-14	--	--	LB01	22009	3252
BT1106	26.868584	89.397420	265	97.3346	1.90E-04	1.63E-14	2.69E-15	--	--	LB_{AVG}	1941	355
BT1107	26.859617	89.465067	349	100.6914	1.89E-04	1.47E-14	1.96E-15	--	--	LB_{AVG}	1665	251
BT1108	26.837079	89.469833	332	94.5717	1.90E-04	4.85E-14	4.59E-15	--	--	LB_{AVG}	6306	620
BT1114	27.033085	90.078891	348	103.8554	1.90E-04	7.39E-14	5.52E-15	--	--	LB_{AVG}	8866	683
BT1116	26.845815	90.183628	335	102.5252	1.89E-04	1.37E-14	2.23E-15	--	--	LB_{AVG}	1511	279
BT1118	26.938299	90.372321	502	100.5142	1.89E-04	1.54E-14	2.23E-15	--	--	LB_{AVG}	1745	284
BT1119	26.941358	90.382716	489	105.7515	1.87E-04	1.62E-14	2.32E-15	--	--	LB_{AVG}	1734	278
BT1125	27.076351	90.761209	628	100.9733	1.89E-04	2.02E-13	9.32E-15	--	--	LB_{AVG}	25060	1192
BT1127	26.965505	90.855892	249	106.9903	1.89E-04	3.02E-14	2.89E-15	--	--	LB_{AVG}	3394	346
BT1128	26.943598	90.872009	222	100.5115	1.84E-04	4.05E-14	2.95E-15	--	--	LB_{AVG}	4764	367
BT1129	26.932064	90.867574	215	104.0817	1.88E-04	3.08E-14	3.66E-15	--	--	LB_{AVG}	3553	446
BT1131	26.858651	90.943339	295	93.4598	1.88E-04	8.67E-14	5.99E-15	--	--	LB_{AVG}	11475	816
BT1133	26.913679	90.910364	211	99.7272	1.88E-04	6.53E-14	5.42E-15	--	--	LB_{AVG}	8031	688
LB01	--	--	--	--	1.94E-04	1.90E-13	2.78E-14	188023	43721	--	--	--
LB04	--	--	--	--	1.84E-04	2.11E-15	8.61E-16	--	--	--	--	--
LB05	--	--	--	--	1.90E-04	5.61E-16	5.37E-16	--	--	--	--	--
LB06	--	--	--	--	1.88E-04	1.12E-15	5.60E-16	--	--	--	--	--
LB07	--	--	--	--	1.90E-04	2.11E-15	8.61E-16	--	--	--	--	--
LB_{AVG}	--	--	--	--	--	--	--	18454	4520	--	--	--

Table S2. Least-squares estimation (LSE) and stream-power model (SPM) statistics. Fixed denotes that the exponent *n* was fixed to a value of 2.2. Note that a *K* values cannot be computed for the SPM using k_{sn}-q data as the SPM only relies on K_{lp} (see equation 4c).

Dataset	C LSE (fixed)	ϕ LSE (fixed)	*K* LSE (fixed)	*n* LSE (fixed)	MSWD LSE (fixed)	MSWD LSE 1σ (fixed)	C LSE (free)	ϕ LSE (free)	*K* LSE (free)	*n* LSE (free)	MSWD LSE (free)	MSWD LSE 1σ (free)	*K* SPM (fixed)	MSWD SPM (fixed)	MSWD SPM 1σ (fixed)
k_{sn} *all data*	14	0.45	3.2E-09	2.2	24.41	0.12	68	0.16	5.6E-18	6.1	21.35	0.12	3.4E-09	24.85	0.12
k_{sn} R = 0.75 m yr^{-1}	19	0.45	1.5E-09	2.2	13.13	0.17	50	0.26	3.4E-13	3.8	12.83	0.17	1.7E-09	13.32	0.17
k_{sn} R = 2 m yr^{-1}	12	0.45	4.5E-09	2.2	17.34	0.28	15	0.40	1.1E-09	2.5	18.60	0.28	4.4E-09	17.47	0.28
k_{sn} R = 3 m yr^{-1}	9.8	0.45	6.5E-09	2.2	23.82	0.30	9.0	0.47	9.1E-09	2.1	24.11	0.30	6.6E-09	23.82	0.30
k_{sn} R = 4.75 m yr^{-1}	7.6	0.45	1.2E-08	2.2	17.09	0.32	119	0.05	2.5E-46	19	25.62	0.32	1.0E-08	16.91	0.32
k_{sn}-q *all data*	18	0.45	1.8E-09	2.2	16.78	0.12	43	0.29	2.6E-12	3.4	15.68	0.12	--	17.78	0.12
k_{sn}-q R = 0.75 m yr^{-1}	21	0.45	1.3E-09	2.2	13.58	0.17	46	0.29	2.0E-12	3.4	12.81	0.17	--	18.83	0.17
k_{sn}-q R = 2 m yr^{-1}	16	0.45	2.4E-09	2.2	16.56	0.28	20	0.41	7.0E-10	2.4	17.41	0.28	--	17.13	0.28
k_{sn}-q R = 3 m yr^{-1}	16	0.45	2.3E-09	2.2	21.32	0.30	12	0.50	6.7E-09	2.0	21.92	0.30	--	21.29	0.30
k_{sn}-q R = 4.75 m yr^{-1}	15	0.45	2.8E-09	2.2	16.52	0.32	231	0.05	4.4E-54	20	26.27	0.32	--	16.53	0.32
